# Non-Descemet stripping endothelial keratoplasty for bullous keratopathy in patients with atopic dermatitis: A long-term case report

**DOI:** 10.1097/MD.0000000000039767

**Published:** 2024-09-27

**Authors:** Saki Sakakura, Risa Yamazaki, Yuichi Uchino, Kazuno Negishi, Shigeto Shimmura

**Affiliations:** a Department of Ophthalmology, Keio University School of Medicine, Shinjuku-ku, Tokyo, Japan; b Department of Clinical Regenerative Medicine, Fujita Medical Innovation Center, Fujita Health University, Ota-ku, Tokyo, Japan

**Keywords:** atopic dermatitis, bullous keratopathy, nDSAEK

## Abstract

**Rationale:**

Patients with atopic dermatitis undergoing penetrating keratoplasty (PKP) face a high risk of postoperative complications. Endothelial keratoplasty may be a safer alternative for such patients, including those with abnormal anterior chamber anatomy.

**Patient concerns:**

3 male patients, aged 33 to 44, presented with blurred vision at Keio University Hospital.

**Diagnosis:**

Bullous keratopathy was diagnosed through slit-lamp examination and specular microscopy. Two patients had well-controlled systemic atopic dermatitis, while 1 had blepharitis associated with atopic dermatitis. Two patients had peripheral anterior synechia, and 2 had undergone glaucoma surgery before keratoplasty.

**Interventions:**

Non-Descemet stripping endothelial keratoplasty (nDSAEK) was performed by a single surgeon.

**Outcomes:**

The best-corrected visual acuity ranged from 0.7 to 1.5 logMAR before surgery and from 0.2 to 2.3 logMAR after surgery. One year post-surgery, the graft remained clear in 2 cases; however, in the case of repeated glaucoma surgeries after nDSAEK, the graft became edematous. Corneal endothelial cell density was 1586 and 1988 cells/mm² in 2 cases and undetectable in the failed case. The follow-up period ranged from 2.5 to 9 years.

**Lessons:**

Despite the presence of peripheral anterior synechia or prior glaucoma surgery, some patients experienced a favorable long-term postoperative course following nDSAEK. This procedure may offer a safer alternative for treating patients with atopic dermatitis who have ocular complications that present a high risk for PKP.

## 1. Introduction

Descemet stripping endothelial keratoplasty (DSAEK) for bullous keratopathy with peripheral anterior synechia or a history of trabeculectomy or glaucoma drainage implant surgery can be complicated, and penetrating keratoplasty (PKP) may be preferred.^[1,2]^ However, patients with atopic dermatitis who undergo PKP are at greater risk of postoperative complications, such as postkeratoplasty atopic sclerokeratitis (PKAS), suture insufficiency, and suture-related infections, than patients without atopic dermatitis.^[3,4]^ This study aimed to report 3 patients with atopic dermatitis who underwent non-Descemet stripping endothelial keratoplasty (nDSAEK) for bullous keratopathy.

## 2. Case presentation

The demographics of all 3 patients are shown in Table 1.

**Table 1:** Patient demographics.

	Case 1	Case 2	Case 3
Age at surgery (yr)	44	42	33
Sex	Male	Male	Male
Atopic dermatitis -Systemic condition -Use of immunosuppressive eyedrops -Blepharitis	Severe − −	Well controlled − −	Well controlled − +
Peripheral anterior synechia	+	−	+
Preoperative glaucoma	+, posttrabeculectomy	+, posttrabeculectomy	+, history of steroid-induced glaucoma, no glaucoma surgery
Period between onset of bullous keratopathy and surgery	4 mo	2 mo	4 mo
Best-corrected visual acuity of the operated eye -Preoperative -1 yr after nDSAEK -Last visit	1.5 logMAR 0.7 logMAR 0.5 logMAR	0.7 logMAR 0.2 logMAR 0.2 logMAR	0.7 logMAR 2.3 logMAR 2.3 logMAR
Intraocular pressure -Preoperative -1 yr after nDSAEK -Last visit	14 mm Hg 10 mm Hg 6 mm Hg	15 mm Hg 9 mm Hg 9 mm Hg	17 mm Hg 20 mm Hg 18 mm Hg
Corneal endothelial specular microscopy -Preoperative -1 yr after nDSAEK -last visit	Undetectable 1988 cells/mm^2^ 1555 cells/mm^2^	Undetectable 1586 cells/mm^2^ 609 cells/mm^2^	Undetectable Undetectable Undetectable
Period between nDSAEK and last visit	9 yr	2.5 yr	3 yr

### 2.1. Case 1

A 44-year-old man was referred to our hospital due to decreased vision in the right eye. The patient underwent phacoemulsification and intraocular lens implantation surgery at the age of 27 years and trabeculectomy at the ages of 41 and 43 years. At the age of 44, bullous keratopathy was diagnosed. He had severe atopic dermatitis treated with oral calcineurin inhibitors; however, blepharitis was not observed, and he had no history of immunosuppressive eye drops.

Initial examination of his right eye revealed the best-corrected visual acuity (BCVA) of 1.5 logMAR and the intraocular pressure of 14 mm Hg with the use of carteolol hydrochloride (Mikelan LA 2%®︎; Otsuka Pharmaceutical Co., Ltd., Tokyo, Japan). Slit-lamp examination revealed bullous keratopathy, a bleb in the upper bulbar conjunctiva, peripheral iridectomy, peripheral anterior synechia, shallow anterior chamber and an intraocular lens. Specular microscopy (EM-3000; Tomey Corp., Nagoya, Japan) results were undetectable. His left eye was 2.8 logMAR due to postoperative glaucoma after phacoemulsification and intraocular lens implantation. The intraocular pressure of the left eye was 16 mm Hg.

**Figure 1.**
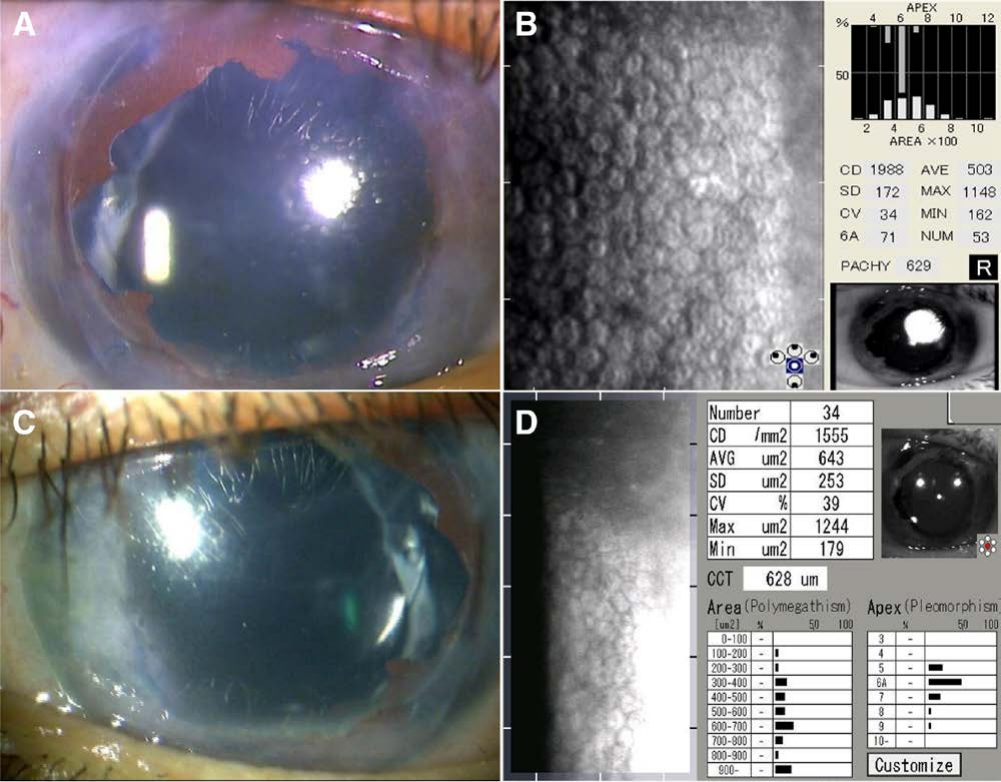
Case 1. Slit-lamp examination and specular microscopy showed the clear graft 1 year (A, B) and 9 years (C, D) after surgery.

He underwent nDSAEK in his right eye 4 months after his initial visit. The postoperative course was uneventful, and 1 year after surgery, the BCVA was 0.7 logMAR, and the intraocular pressure was 10 mm Hg. The cornea was clear upon slit-lamp examination, with the corneal endothelial cell density of 1988 cells/mm^2^ (Fig. 1A,B). Nine years after surgery, the BCVA was 0.5 logMAR, and the corneal endothelial cell density was 1555 cells/mm^2^ with a clear cornea (Fig. 1C,D).

### 2.2. Case 2

A 40-year-old man was referred to our hospital because of decreased vision in his left eye. The right eye was enucleated after vision loss due to proliferative vitreous retinopathy. At the ages of 23, 26, and 28 years, he underwent trabeculectomy for secondary glaucoma in his left eye. At the age of 27, phacoemulsification and intraocular lens implantation were performed. At the age of 37 years, bleb needling was performed, and 3 years later, he was diagnosed with bullous keratopathy. The patient exhibited mild atopic dermatitis but had no history of severe blepharitis or immunosuppressive eye drop use.

**Figure 2.**
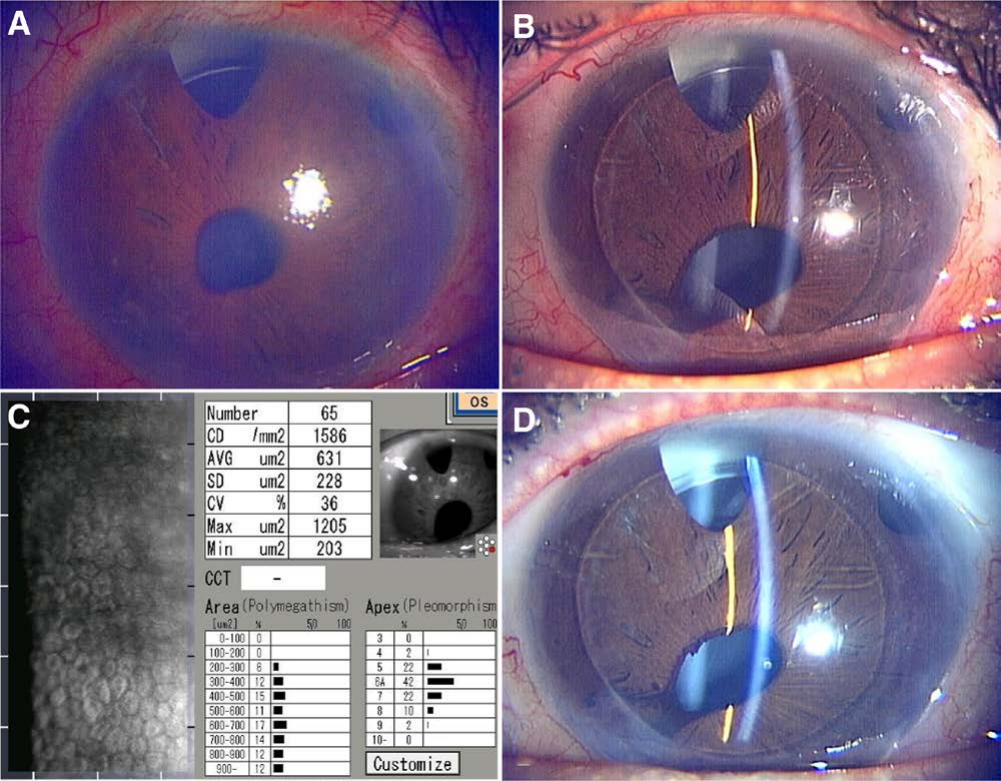
Case 2. (A) Slit-lamp examination showed mild bullous keratopathy with 2 blebs in the upper bulbar conjunctiva prior to nDSAEK. (B, C) Clear donor graft 1 year after nDSAEK with specular microscopy. (D) Clear donor graft after 2 and a half years after nDSAEK.

The extent of bullous keratopathy in the left eye was not severe at the initial presentation. At the age of 42, he showed deterioration of his visual acuity. The BCVA at that time was 0.7 logMAR, and the intraocular pressure was 15 mm Hg with travoprost/timolol malate (Duotrav®︎; Novartis Pharma, Basel, Switzerland). Slit-lamp examination revealed mild bullous keratopathy with 2 blebs in the upper bulbar conjunctiva, peripheral iridectomy, and an intraocular lens (Fig. 2A). Specular microscopy results were undetectable. Two months after vision impairment in the left eye, the patient underwent nDSAEK without any complications. Bleb revision was performed 5 months after nDSAEK. One year after nDSAEK, the BCVA was 0.2 logMAR, and the intraocular pressure was 9 mm Hg. Slit-lamp examination revealed a clear cornea, and specular microscopy revealed 1586 cells/mm^2^ (Fig. 2B,C). Two and a half years after surgery, BCVA remained at 0.2 logMAR, and specular microscopy revealed 609 cells/mm^2^ with a clear cornea (Fig. 2D).

### 2.3. Case 3

A 33-year-old male patient with decreased vision in the right eye presented at our hospital. At the age of 15 years, phacoemulsification and intraocular lens implantation were performed in the right eye. Secondary glaucoma was diagnosed at the age of 23 years. At the age of 33, he was diagnosed with bullous keratopathy. His left eye was pseudophakic with a clear cornea. Systemic symptoms of atopic dermatitis were self-limiting, and he did not use immunosuppressive eye drops.

**Figure 3.**
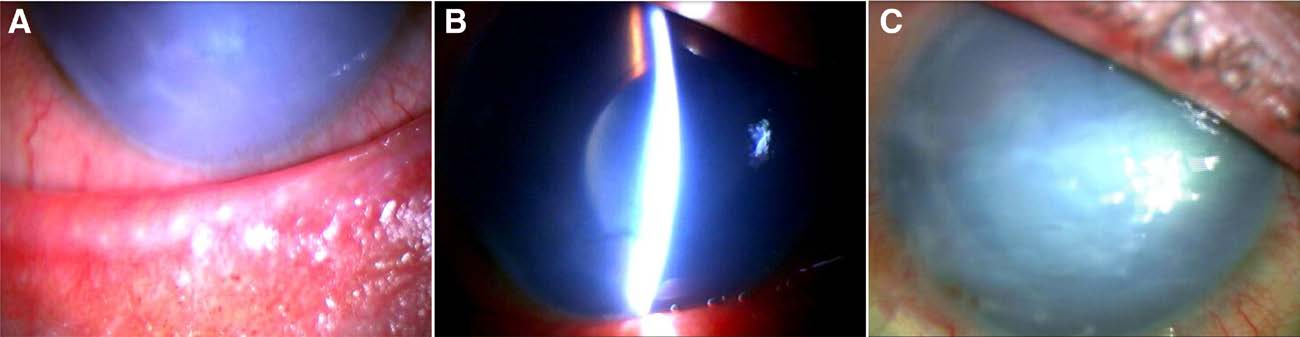
Case 3. (A) Slit-lamp examination showed blepharitis prior to surgery. (B) Bullous keratopathy, peripheral anterior synechia and shallow anterior chamber was observed prior to nDSAEK. (C) Bullous keratopathy 1 year after nDSAEK.

The BCVA in his right eye was 0.7 logMAR, and the intraocular pressure was 17 mm Hg with dorzolamide hydrochloride/timolol malate (Cosopt®︎; Santen Pharmaceutical Co., Ltd., Osaka, Japan) and tafluprost (Tapros®︎; Santen Pharmaceutical Co., Ltd.). In his left eye, the BCVA was 0.1 logMAR, and his intraocular pressure was 8 mm Hg. Slit-lamp examination revealed atopic blepharitis in both eyes, moderate bullous keratopathy in his right eye with corneal neovascularization, peripheral anterior synechia, shallow anterior chamber, and an intraocular lens (Fig. 3A,B). Four months after visual blurring, the patient underwent nDSAEK. The immediate postoperative corneal condition was uneventful; however, 1 week after nDSAEK, the intraocular pressure increased to 35 mm Hg according to the Goldmann applanation tonometer. Although he had a history of steroid-induced ocular hypertension, treatment with 0.1% betamethasone (Sanbethasone®︎; Santen Pharmaceutical Co., Ltd.) 5 times daily was continued to prevent graft failure. The intraocular pressure remained high with dorzolamide hydrochloride/timolol malate (Cosopt®︎; Santen Pharmaceutical Co, Ltd.), tafluprost (Tapros®︎; Santen Pharmaceutical Co, Ltd.), brimonidine tartrate (Aiphagan®︎; Senju Pharmaceutical Co, Ltd., Osaka, Japan), and oral acetazolamide. Therefore, trabeculectomy and Baerveldt glaucoma device implantation (BG101-350; Johnson & Johnson, Tokyo, Japan) were performed 2 and 6 months after nDSAEK, respectively. One year after surgery, the BCVA was 2.3 logMAR, and the intraocular pressure was 20 mm Hg with dorzolamide hydrochloride/timolol malate (Cosopt®︎; Santen Pharmaceutical Co., Ltd.) and tafluprost (Tapros®︎; Santen Pharmaceutical Co., Ltd.). Slit-lamp examination revealed severe bullous keratopathy, and specular microscopy was undetectable (Fig. 3C). Treatment with betamethasone (0.1%) was continued for 21 months after nDSAEK and was then stopped. Three years after nDSAEK, the visual acuity and slit-lamp examination findings were similar to those at 1 year after nDSAEK.

## 3. Discussion

DSAEK can be challenging in eyes with peripheral anterior synechia, a history of trabeculectomy or the implantation of glaucoma drainage devices. Shallow anterior chamber also makes DSAEK difficult and may induce pupillary block.^[1]^ In cases of posttrabeculectomy or postglaucoma drainage implant surgery, poor graft adherence can occur if air escapes to the filtering bleb or glaucoma drainage implant.^[1,2,5]^ Graft dislocation is also possible if a patient develops hypotony.^[6]^ PKP may be a safer option for such patients, such as the 3 patients included in our study. However, PKP for atopic dermatitis patients can cause complications such as PKAS, suture insufficiency, and infection of the suture, which may lead to graft failure.^[3,4,7]^

PKAS is a severe type of sclerokeratitis that typically develops within 1 to 4 weeks of keratoplasty. The findings include early loosening of sutures and persistent epithelial defects associated with symptoms of photophobia, epiphora, hyperemia, and eye pain.^[3]^ Blepharitis is a risk factor for PKAS. Its incidence was reported to be 17% among atopic dermatitis patients,^[8]^ although ocular complications are not included in the severity scoring of atopic dermatitis index.^[9]^ In this study, the systemic condition was self-limiting in 2 patients at the time of nDSAEK, however, all 3 patients had a history of severe atopic dermatitis with secondary cataract. One patient had atopic blepharitis, which alone was a reason for avoiding PKP.

Another well-known complication of atopic dermatitis following PKP is bacterial and viral infections.^[4,10]^ A prior study revealed that, irrespective of the severity of atopic keratoconjunctivitis and facial dermatitis, bacterial detection rates were significantly greater in patients with atopic dermatitis, with approximately 85% detection in atopic patients compared to 25% in normal volunteers.^[11]^ In addition, DSAEK has a significantly lower rate of postoperative microbial keratitis than PKP with less use of steroids.^[12]^ Reported predisposing factors for bacterial infection include glaucoma treatment and lid abnormalities such as blepharitis.^[12,13]^ In this study, all 3 patients used antiglaucoma eye drops, and 1 patient had blepharitis, which was another reason for avoiding PKP.

To avoid complications associated with PKP in atopic patients, we performed nDSAEK despite the presence of risk factors such as peripheral anterior synechia, shallow anterior chamber, and previous trabeculectomy. Fortunately, no intraoperative or immediate postoperative complications, such as graft dislocation or poor graft adherence, were observed. Two of the 3 patients showed a good postoperative course for more than 1 year after nDSAEK; however, 1 patient presented with early graft failure. Graft failure is thought to be caused by prolonged postoperative high intraocular pressure. In general, DSAEK may be less likely to cause postoperative ocular hypertension or glaucoma than PKP, which is reported to occur in 18% of DSAEK patients and 27% of PKP patients.^[14]^ One of the main reasons for postoperative glaucoma after DSAEK is steroid-induced high intraocular pressure,^[15,16]^ which typically develops 4 to 6 weeks after initiating steroid eye drops but can develop as early as 1 week after DSAEK.^[17,18]^ A previous report showed that postoperative glaucoma can be controlled by lowering the steroid concentration earlier than PKP, suggesting that DSAEK may be a good choice for glaucoma patients.^[18]^ Although a regimen of steroid tapering for steroid-induced glaucoma after DSAEK has not been established, switching from dexamethasone 1% to loteprednol etabonate during 6 months postoperatively or to fluorometholone 0.1% after 6 months postoperatively has been reported.^[18]^ In case 3, given the severe blepharitis and history of steroid-induced high intraocular pressure, we chose DSAEK rather than PKP to reduce the risk of postoperative complications. Immediately after nDSAEK, the intraocular pressure was normal without pupillary block, but he experienced high intraocular pressure 1 week after nDSAEK. Among the reported risk factors for ocular hypertension after keratoplasty, this patient had multiple factors, such as steroid-induced ocular hypertension, preexisting glaucoma with intraocular pressure > 16 mm Hg, pseudophakia, and peripheral anterior synechia.^[1,15,18]^ However, other patients with a good postoperative course also had preexisting glaucoma, pseudophakia, and peripheral anterior synechia. Therefore, steroid-induced ocular hypertension may be the main cause of postoperative glaucoma.^[15,16]^

In conclusion, we reported 3 cases of nDSAEK in patients at risk of developing PKP-related complications due to atopic dermatitis. Although patients had peripheral anterior synechia or previous glaucoma surgery, which are risk factors for DSAEK, 2 of the 3 patients in this study had a good postoperative course. Although the number of cases presented in this report is limited, our findings suggest that DSAEK may serve as a safer alternative for treating atopic dermatitis patients with ocular complications that present a high risk for PKP.

## Author contributions

**Conceptualization:** Shigeto Shimmura.

**Data curation:** Saki Sakakura, Risa Yamazaki, Shigeto Shimmura.

**Supervision:** Kazuno Negishi, Shigeto Shimmura.

**Writing – original draft:** Saki Sakakura.

**Writing – review & editing:** Risa Yamazaki, Yuichi Uchino, Shigeto Shimmura.
